# Action Intentions Modulate Allocation of Visual Attention: Electrophysiological Evidence

**DOI:** 10.3389/fpsyg.2012.00379

**Published:** 2012-10-04

**Authors:** Agnieszka Wykowska, Anna Schubö

**Affiliations:** ^1^General and Experimental Psychology Unit, Department Psychologie, Ludwig-Maximilians-Universität in MunichMunich, Germany; ^2^Fachbereich Psychologie, Philipps-UniversitätMarburg, Germany

**Keywords:** action-perception links, intentional weighting, visual attention, ERPs

## Abstract

In line with the Theory of Event Coding (Hommel et al., 2001), action planning has been shown to affect perceptual processing – an effect that has been attributed to a so-called *intentional weighting* mechanism (Wykowska et al., 2009; Hommel, 2010). This paper investigates the electrophysiological correlates of action-related modulations of selection mechanisms in visual perception. A paradigm combining a visual search task for size and luminance targets with a movement task (grasping or pointing) was introduced, and the EEG was recorded while participants were performing the tasks. The results showed that the behavioral congruency effects, i.e., better performance in congruent (relative to incongruent) action-perception trials have been reflected by a modulation of the P1 component as well as the N2pc (an ERP marker of spatial attention). These results support the argumentation that action planning modulates already early perceptual processing and attention mechanisms.

## Introduction

Being active agents in the world, humans must have developed means to optimize their interaction with the environment through efficient action planning. What does action planning consist in? Several researchers postulate that actions are represented as action goals and these, in turn, are represented as sensory effects of planned actions (e.g., James, 1890; Greenwald, 1970; Prinz, 1987, 1997; Hommel et al., 2001). Imagine you are planning to hit a tennis ball with your racket. Your brain presumably represents that action in the form of a somatosensory feedback of how it will feel on your arm to hit the object with a given force. According to Hommel et al. (2001), the action representation will also involve more “distal” sensory effects, such as visual perception of a motion trajectory of the hit ball as well as the sound of the ball struck by the racket. Such a way of representing planned action might indeed prove efficient, as it entails that consequences of actions which do not match expected effects need to be corrected. Humans must, therefore, learn given consequences of their actions through life-long experience with those actions (e.g., Hommel, 2010). Similar ideas are also implemented in forward models of motor control (e.g., Wolpert and Ghahramani, 2000).

### Common-coding of action and perception characteristics

If actions are represented in form of sensory consequences of the planned actions, action planning and perception need to be tightly coupled (e.g., Prinz, 1997; Hommel et al., 2001). The idea of close action-perception coupling is in line with ideomotor views (e.g., James, 1890; Greenwald, 1970; Prinz, 1987, 1997; Hommel et al., 2001) as well as common-coding perspectives, such as the Theory of Event Coding (TEC: Prinz, 1987, 1997; Hommel et al., 2001) that clearly speak against traditional views postulating linear stage models of processing (e.g., Sternberg, 1969; for a discussion see Hommel et al., 2001). Such traditional views state that processing takes place in sequential stages, i.e., for example, perceptual processing, memory, action selection, action execution that can be studied autonomously without taking other stages – especially the later ones – into account.

In the theoretical framework proposed in TEC, and in line with earlier ideomotor perspectives, perception, and action share a common representational code, which allows for efficient action planning. This common code consists in a network of features distributed across domains (such as action or perception) that can be bound together to represent common sensorimotor events.

A common code implies bi-directional links between action and perception. Such links and mutual influences have been supported by a growing body of empirical evidence, where the findings showed interference effects in situations when a code for action and perception has been occupied and needed updating (e.g., Müsseler and Hommel, 1997; Hommel, 1998).

Evidence for close coupling between action and perception has been brought forward also by imaging techniques. For example, Schubotz and von Cramon (2002) carried out a series of fMRI-studies in which sequences of stimuli were presented. The data showed that when participants were judging whether certain sequences of stimuli are in accordance with a rule (either increasing size of visually presented disks or increasing pitch of a sequence of auditory tones) the respective areas of premotor cortex were activated: that is, hand-related areas were activated when the rule was related to the size of the disks and articulation areas were activated when the rule was related to tone pitch. These results showed an automatic activation of motor areas when action-relevant perceptual attributes were processed, speaking in favor of strong action-perception coupling.

Similarly, Grèzes and Decety (2002) or Grafton et al. (1997) showed automatic activation of motor areas when objects bearing certain affordances (Gibson, 1977) were only viewed. Studies by Kiefer and colleagues using an action priming paradigm have also shown that perceptual processes such as object recognition can be modulated by action-object congruency (e.g., Helbig et al., 2006, 2010), and that these effects may be rather early in perceptual processing (Kiefer et al., 2011; see also Humphreys et al., 2010). Moreover, Tucker and Ellis (2001) observed the effects of object affordances on motor responses in a visual categorization task. In their study, participants were asked to discriminate objects as being artificial (e.g., hammer, nail) or natural (e.g., cucumber, grape). Participants responded with either a power- or precision grip dependent on the category of objects (artificial vs. natural). Size of objects was completely irrelevant and orthogonal to the task. Yet, precision responses were facilitated if the object was smaller and power grips were made faster in response to larger objects. Results of the study by Tucker and Ellis have been interpreted in line with the idea of object affordances (Gibson, 1977), which, even if irrelevant to the task, activate certain motor responses that would be compatible with the object properties. Consequently, if a required action is incongruent with the afforded one, impaired performance is observed, relative to congruent scenarios.

The concept of affordances not only implies automatic activation of a motor program through perceiving action-affording objects but can also have consequences in opposite direction, i.e., action-related bias on attentional processes. Evidence for the latter has been found in neuropsychological case studies (e.g., Humphreys and Riddoch, 2001; di Pelligrino et al., 2005). In the study of Humphreys and Riddoch (2001), a patient suffering from visual extinction was better in detecting objects on the neglected side when the objects were defined by their action affordances, as compared to other characteristics. di Pellegrino et al. (2005) reported that visual extinction patients showed a behavioral benefit for the extinction site when the presented objects had characteristics affording an action on that site (e.g., a cup with a left handle). Another piece of evidence for a bias of spatial attention through action-affording characteristics of perceived objects has been brought forward by an ERP/fMRI study of Handy et al. (2003), in which a sensory ERP component (P1) has been modulated by (implicit) action-relevance of stimuli. Pictorial action-congruency effects were also reported in a recent study by Kiefer et al. (2011), where ERP-modulations in the P1 latency range were observed for stimuli that afforded the same action as an earlier presented prime. These effects were, however, prominent over central electrode sites and were related to activity of motor areas.

### Intentional weighting mechanism and attentional selection

The above-described studies focused mainly on the evidence for a close coupling between action and perception based on the concept of affordances. However, this concept does not determine the underlying mechanism of the observed action-perception coupling. If spatial attention is biased with respect to action-related attributes of the environment, then what sort of mechanism is employed by the brain to impose such a bias? A postulate of a common code for action and perception implies similar selection mechanisms in both domains.

Research in the area of visual attention has established that attentional selection is a result of a biased competition (e.g., Bundesen, 1990; Desimone and Duncan, 1995; Reynolds et al., 1999) or weighted processing of perceptual features and/or dimensions (e.g., Wolfe, 1994; Müller et al., 2003, 2009; Wolfe et al., 2003). If action and perception share a common code, then similar weighting mechanism should operate with respect to action planning. This has indeed been postulated through the idea of the *intentional weighting* mechanism (Hommel et al., 2001; Hommel, 2010). According to the authors, the intentional weighting mechanism prioritizes processing of those perceptual characteristics that are relevant for intended actions. Hommel (2010) claims that such a mechanism has developed in order to provide information for open parameters of online action control. However, once it developed to serve such a function, it became also available for other processes, also in the absence of planning of overt action.

Craighero et al. (1999) observed effects that might be interpreted in line with such an idea of an intentional weighting mechanism: in their study, latencies of a grasping movement toward a particular object were reduced when a visually presented go-signal was congruent with to be grasped object (a left- or right- oriented bar). Craighero et al. concluded that planning a given action (e.g., grasping) biased visual detection (of the go-signal).

Fagioli et al. (2007) directly tested the idea of intentional weighting, using an oddball paradigm in which a sequence of stimuli was presented on a computer screen. The oddballs were either size or location oddballs and were to be detected. At the same time, participants were asked to either grasp a white cube or point toward a white dot. The authors found that when participants were preparing for a grasping movement they detected size oddballs faster than luminance oddballs whereas location oddballs were detected faster than size oddballs in the pointing condition. The authors concluded that perceptual dimensions were weighted with respect to action planning, which resulted in such differential pattern of behavior.

Wykowska et al. (2009) conducted a series of experiments along similar lines. In this series, a more classical attention task (a visual search task) was used to investigate whether intentional weighting modulates visual attention. The paradigm consisted of two tasks: a visual search for size or luminance pop-out targets presented on a computer screen, and a movement task: pointing or grasping of items placed on an especially designed device below the computer screen. Importantly, the two tasks were completely unrelated both perceptually (different objects to be detected in the visual search task and different objects to be grasped/pointed to), and motorically: the visual search task was performed with mouse key presses with the dominant hand (target present: one key vs. target absent: the other key) whereas the grasping/pointing action was performed with the non-dominant hand on the items of the device (for details of the design, see Wykowska et al., 2009). The authors observed that size detection was better when participants were preparing for a grasping action (*congruent* condition) as compared to pointing (*incongruent* condition) whereas luminance detection was improved when participants were preparing for a pointing movement (*congruent* condition), relative to grasping (*incongruent* condition). The authors termed these effects action-perception *congruency* *effects* as they manifested the idea of facilitated processing for congruent pairs (e.g., grasping and size) relative to incongruent pairs (e.g., grasping and luminance). Similarly to Fagioli et al. (2007), Wykowska et al. concluded that processing of perceptual dimensions seem to be biased (weighted) by action planning – thanks to the *intentional weighting* mechanism (e.g., Hommel, 2010; Memelink and Hommel, 2012). Importantly, Wykowska et al. (2009) observed that such a bias can already be observed at early stages of processing that are manifested in a simple task of search for pop-out. The authors concluded that action planning might be another source of a top-down control over bottom-up perceptual processing in a similar way as a task-related weighting mechanism weighs task-relevant perceptual dimensions higher than the irrelevant dimensions (e.g., Müller et al., 2009).

### Aim of the present study

The aim of the present study was to investigate the intentional weighting mechanism with the EEG/ERP methodology. Attentional theories (Wolfe, 1994; Müller et al., 1995, 2003) postulate that processing certain characteristics of the environment can be weighted pre-selectively, and that this weighting affects visual attention. If so, then intentional weighting (given that it is similar to other weighting mechanisms) should influence attentional selection processes. In order to test this, we used the ERP technique, which allows focusing on the correlates of attentional selection (the N2pc component) and early sensory pre-selective processes (P1 or N1 components).

Wykowska et al. (2009) suggested that perceptual *dimensions* are weighted with respect to action planning. Hence, intentional weighting should occur pre-selectively (e.g., Müller et al., 2003), and through pre-selective bias that should influence attentional focus. If that were to be the case, then early sensory ERP components, such as P1 and/or N1, around the time window of 100 ms post-stimulus, should be modulated by action intentions. Although P1 and N1 components are traditionally interpreted as reflecting effects of spatial attention (e.g., Luck et al., 1993; Luck and Hillyard, 1995; Hillyard et al., 1998; Hopfinger and Mangun, 1998; Wykowska and Schubö, 2010, 2011), recent data suggest that P1/N1 components might also reflect a biasing mechanism that operates at the early level of feature/dimension weighting, not necessarily being restricted to *spatial* attention (see Zhang and Luck, 2009 for a discussion on feature-based attention effects on P1). Hence, we hypothesized that pre-selective weighting of dimensions should be observable at early stages of processing (as reflected by the P1/N1 components), i.e., before attention allocation (as reflected by the N2pc).

At the same time, however, such a weighting mechanism should also affect focal attention. It is postulated (Müller et al., 2003; Wolfe et al., 2003) that in a visual search for a feature target, attention is allocated to a location on the basis of a master map of activity that exhibits the highest signal. This signal is a result of a weighted sum of signals coming from various dimension maps. To be more specific, if there is a size pop-out target in the visual field, a strong signal will be elicited in the size dimension map. A weighting mechanism might modulate this signal – either decrease or increase it, dependent on the relevance of the given dimension. In effect, deployment of attention to a location on a master map can be modulated accordingly. Therefore, if action planning weighs perceptual dimensions in a similar manner (Wykowska et al., 2009), it might result in modulation of not only early stages of processing, as reflected by the P1/N1 ERP components, but as a consequence, also an attention-related ERP component, namely, the N2pc. The N2pc is measured at posterior sites within the time window of ca. 180–300 ms and is more negative on contralateral electrode sites compared to ipsilateral electrode sites relative to an attended object presented in the left or right visual hemifield (e.g., Luck and Hillyard, 1994; Eimer, 1996). Although it is not entirely clear whether N2pc reflects a filtering process in the presence of distractors (Luck and Hillyard, 1994) or attentional selection process *per se*, i.e., enhanced processing even in the absence of distractors (Eimer, 1996), N2pc is generally assumed to reflect deployment of attention to objects in the visual field (Eimer, 1996; Woodman and Luck, 2003; Luck, 2005; Jolicoeur et al., 2006). Therefore, observing action-related modulation of the N2pc would indicate that the focal attention is biased by action planning, presumably due to a weighting mechanism that operates at perceptual dimensions.

To meet the aim of the present study, we introduced a paradigm similar to the experimental design of Wykowska et al. (2009). Participants had to perform a visual search task for size and luminance targets and responded with the dominant hand on mouse keys. Additionally, participants were asked to perform a grasping or pointing action (with the other hand) on three linearly aligned cups positioned under the computer screen (as Wykowska et al. (2011) have shown, the congruency effects can be observed even with completely reduced perceptual similarity between action and perception contexts). With the two types of target dimensions (size vs. luminance) and two types of actions (grasping vs. pointing) we created two action-perception congruency pairs (in line with Wykowska et al., 2009, 2011). That is, size was assumed to be a congruent dimension for grasping (during grasping one needs to specify size of grip aperture, among other parameters) and luminance was assumed to be a relevant dimension for pointing (luminance targets enable efficient localization of an object with a pointing movement response (e.g., Graves, 1996; Anderson and Yamagishi, 2000). While participants were performing the task, the EEG signal was recorded. We expected to replicate the behavioral results of Wykowska et al. (2009) and hypothesized that the congruency effects should be observed in the form of modulation of either the P1/N1 ERP complex, the N2pc, or both.

## Materials and Methods

### Stimuli and apparatus

**Figure 1:**
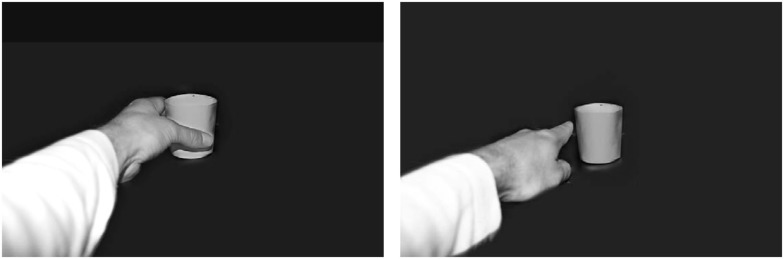
**Movement cues: grasping movement cue (left) and pointing movement cue (right)**. The cues were made to be as physically similar as possible, so that they would not elicit different brain response related to their physical characteristics.

Stimuli were presented on a 17-inch computer screen with a 100 Hz refresh rate placed at a distance of 100 cm from an observer. The movement cues consisted in black-and-white pictures of a left hand grasping or pointing to a paper cup (Figure 1) presented in the middle of the computer screen covering 11.8° × 17.7° of visual angle.

**Figure 2:**
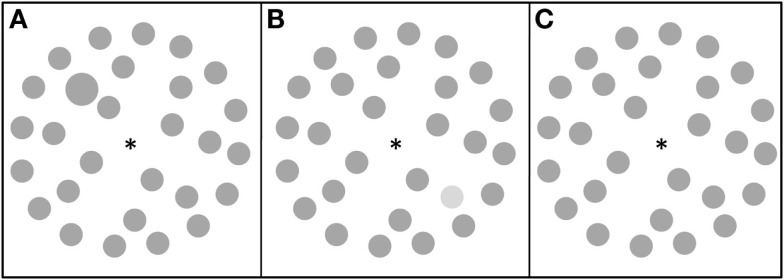
**Visual search stimuli: (A) a size target display; (B) a luminance target display; (C) a blank display**.

The items of the search display were positioned on three imaginary circular arrays with diameters of 4.2°, 9.9°, and 15.3° of visual angle on a light-gray background. Sixteen elements were positioned on the outermost circle; eight elements were presented on the middle circle; and four elements on inner circle. All elements were dark gray (22 cd/m^2^) except for the luminance target (53 cd/m^2^). Size of elements covered 1.5° of visual angle in diameter, except for the size target, which was larger: 2° of visual angle. There were two possible display types: a target present display (50% of trials), Figures 2A,B; and a blank display, Figure 2C. The target could appear at one of six positions (upper/middle/lower and left/right to the fixation point, on the middle circular array).

**Figure 3:**
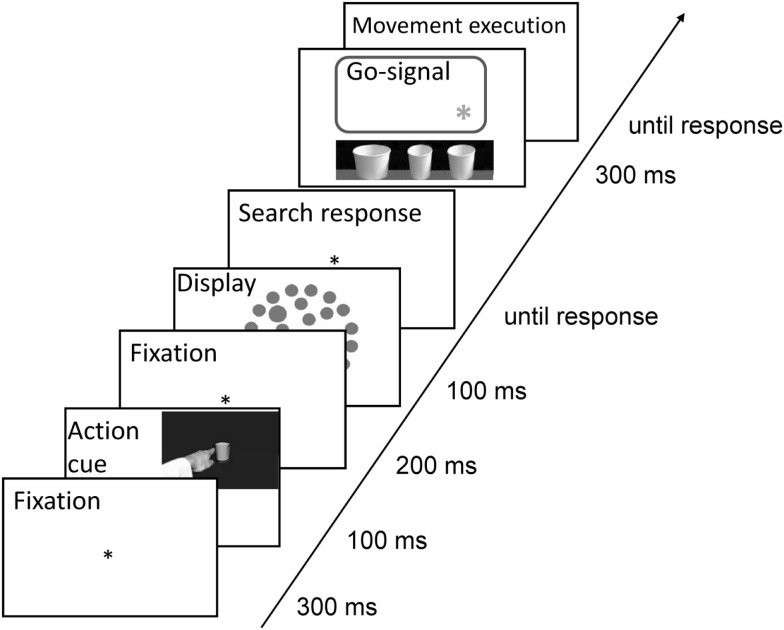
**Trial sequence**. First, a movement cue was presented. Participants were asked to only prepare for the movement but not execute it at this stage. Subsequently, after a short presentation of a display with fixation asterisk, a visual search display was presented. Participants were asked to respond to the search task immediately and be as fast and as accurate as possible. Upon completion of the search task, a yellow asterisk presented on the screen signaled which of the three cups placed below the computer screen in a horizontal line should be grasped or pointed to (dependent on the cue presented at the beginning of the trial). Only at this point, participants executed the prepared movement. In this task, accuracy, but not speed was stressed.

The go-signal for movement execution consisted in a yellow asterisk of 0.6° in diameter, CIE L*a*b color coordinates: 87/5/82. It was presented 4.5°, 11.3°, or 17.7° from the left border of the screen signaling the to be grasped/pointed to paper cup situated beneath the computer screen, each cup being situated directly below one of the asterisk positions, see Figure 3.

The to be grasped/pointed to cups were placed on a table below the computer screen 70 cm in front of the observers, to allow for easy reach. There were three cups: a small white (3 cd/m^2^) cup, 5 cm (2.8°) in diameter in the middle point; a middle gray (1.8 cd/m^2^) cup, 6.5 cm (3.7°) in diameter in the middle point; and a large dark gray (0.43 cd/m^2^) cup, 8 cm (4.5°) in diameter in the middle point. They were all equal in height (4.5°) and weight (2 g).

### Participants

Eighteen participants (13 women) aged from 21 to 30 years (mean age: 24.3) took part; 8 participants took part in the experiment for course credit, 10 were paid volunteers. Five participants were left-handed, all had normal or corrected to normal vision. Visual acuity was tested with a Rodenstock R12 vision tester (stimuli 112). The experiment was conducted with the understanding and consent of each participant. None of the observers had taken part in an experiment with such a paradigm before.

### Procedure

A trial started with a 300 ms fixation display (a black asterisk of 0.5 cm in diameter in the center of the screen). Subsequently, a movement cue was presented for 100 ms (see Figure 3) followed by another fixation display presented for 200 ms. Next, a search display was presented for 100 ms. Upon response to the search task and a blank screen (400 ms), the go-signal asterisk was presented for 300 ms. The asterisk indicated which of the three cups should be grasped/pointed to. At this point, participants executed the prepared movement, which was registered by an experimenter (who observed performance with a camera outside of the chamber) with a mouse key press. Following the experimenter’s button press, a blank screen was presented for 100 ms, which constituted the inter-trial interval.

In order to be able to perform a subtraction of ERP potentials and extract only search-locked ERPs without the overlapping cue-locked ERPs, catch trials were introduced in the design (30% of all trials, randomly intermixed with standard trials). These differed from the standard trials only in that in place of a search display, another fixation display was presented for 100 ms. As participants did not need to perform a search task, a blank display was presented for 500 ms during the time they would respond to the search display in case of trials of interest. The rest of the trial following the blank display was identical to the actual trials of interest.

Response assignment in the search task was counterbalanced, participants were asked to press one of the mouse keys for target present, the other for target absent, with index and middle fingers of their right hand. Speed and accuracy was stressed in the search task whereas only accuracy was stressed in the movement task.

There were altogether 504 trials for each of the tasks. The target type (size or luminance) was blocked (task order was counterbalanced across participants), whereas the movement type (grasp vs. point) and display type (target present vs. blank) were randomized within a block. Short breaks were introduced after each 63 trials so that participants could move their eyes, blink, and relax. Otherwise, participants were asked to reduce blinking and movement not to introduce excessive movement and eye artifacts.

Before the experimental session proper, participants took part in a practice session (without EEG recording) on a separate day, in which they practiced first only the movement task, without the visual search task, and then 270 regular trials for each of the target type (size vs. luminance). The practice session was scheduled minimum 1 day and maximum 2 days before the experimental session proper. During the experimental session, before the actual start of the experiment, participants did 18 warm-up trials with movement only and 18 trials with search + movement.

### EEG recording

EEG was recorded with Ag-AgCl electrodes from 37 electrodes (Fp1, Fp2, F3, F4, Fz, F7, F8, F9, F10, FC1, FC2, FC5, FC6, C3, C4, CP1, CP2, CP5, CP6, T7, T8, TP9, TP10, P3, P4, Pz, P7, P8, PO3, PO4, POz, PO7, PO8, O1, O2, Oz, VEOG). The electrodes were mounted on an elastic cap (EASYCAP, GmbH, Germany), according to the International 10-10 System. Horizontal and vertical EOG were recorded bipolar from the outer canthi of the eyes and from above and below the observer’s left eye, respectively. All electrodes were referenced to Cz and re-referenced offline to the average of all electrodes. Electrode impedances were kept below 5 kΩ. Sampling rate was 500 Hz with a High-Cutoff Filter of 125 Hz.

### Data analysis

#### EEG data

EEG was averaged offline over 600-ms epoch including a 200-ms pre-stimulus baseline with epochs time locked to search display onset. Trials with eye movements and blinks on any recording channel (indicated by any absolute voltage difference in a segment exceeding 80 μV or voltage steps between two sampling points exceeding 50 μV) were excluded from analyses. Additionally, channels with other artifacts were separately excluded if amplitude exceeded ±80 μV or any voltage was lower than 0.10 μV for a 100 ms interval. Raw data was filtered offline 40-Hz high-cutoff filter (Butterworth zero phase, 24 dB/Oct). Only trials with correct movement and correct search responses were analyzed. Responses in the search task deviating more than ±3 SD from mean RT (calculated separately for each participant and target type) were categorized as outliers and excluded. One participant was excluded from analyses due to extensive eye blinks, two due to extensive alpha waves and one due to poor performance in the movement task (14% of errors in the pointing condition; other participants did not exceed 7%). The analyses focused on O1, O2, PO7, PO8 electrodes, where early visual processing is most pronounced.

#### Behavioral data

Error rates were computed for each participant in both the search task and the movement task. Similarly as in the case of EEG data analysis, prior to RT analysis in the search task, errors in any of the two tasks as well as outliers in the search task were excluded (±3 SD from mean RT for each participant and each target type separately). Error rate analyses in the search task were conducted on correct movement trials. Participants excluded from the EEG data analyses were also excluded from the behavioral analyses.

## Results

### Behavior

#### Reaction times

**Figure 4:**
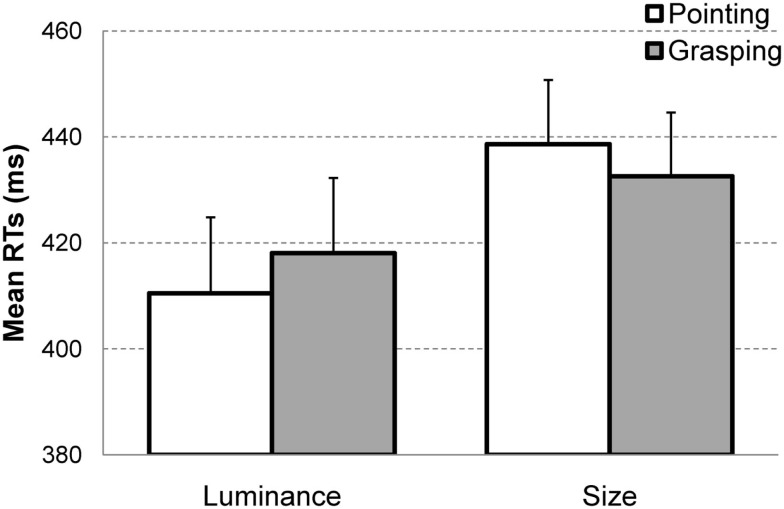
**Mean reaction times (RTs) in target present trials as a function of task type (luminance or size) and movement type (pointing or grasping)**. The *congruency effect* consists in shorter RTs for the congruent action-perception pairs, i.e., size-and-grasping and luminance-and-pointing as compared to incongruent pairs, i.e., size-and-pointing and luminance-and-grasping. Error bars represent the standard errors of the mean.

A 2 × 2 × 2 analysis of variance (ANOVA) on mean RTs with the within-subject factors display type (target present vs. target absent), task type (size vs. luminance), and movement type (pointing vs. grasping) as well as order (size first vs. luminance first) as between-subjects factor showed a main effect of task type, *F*(1, 12) = 16.2, *p* < 0.005, $\eta_{\text{P}}^{2}=0.57$ indicating faster RTs in the luminance task (*M* = 419 ms, SEM = 16) relative to the size task (*M* = 436 ms, SEM = 15). This effect did not interact with order, *p* > 0.5. The main effect of order also did not reach significance, *p* > 0.7. Most importantly for the purposes of this experiment, the interaction of display type, task type and movement type was significant, *F*(1, 12) = 6, $p<0.05,\;\eta_{\text{P}}^{2}=0.33$. This interaction reflected the congruency effect for target present trials: when participants searched for size targets, performance was faster in the grasping condition (*M* = 433 ms, SEM = 12) relative to pointing (*M* = 439 ms, SEM = 12) whereas in search for luminance targets, the effects were in the opposite direction, i.e., pointing condition yielded faster RTs (*M* = 410 ms, SEM = 14) than grasping (*M* = 418 ms, SEM = 14), see Figure 4. This effect did not interact with the order factor, *p* > 0.8.

Subsequent analyses conducted for target present and absent trials separately showed that the interaction of movement type and task type was significant for target present trials *F*(1, 12) = 16, *p* < 0.005, $\eta_{\text{P}}^{\text{2}}=\text{0}\text{.58,}$ but not for target absent trials, *p* > 0.7 (Luminance task, pointing: *M* = 422 ms; SEM = 20 vs. grasping: *M* = 425; SEM = 20; Size task, pointing: *M* = 433 ms; SEM = 19 vs. grasping: *M* = 439 ms; SEM = 17). In neither target present or absent trials, was the interaction of task type and movement type modulated by order, both *p* > 0.5.

Finally, planned comparisons between grasping and pointing conditions for size and luminance tasks separately (target present trials) revealed that the difference between those two conditions was significant in the luminance task, *t*(13) = 2.1, *p* < 0.05 (one-tailed) and marginally significant in the size task, *t*(13) = 2.1, *p* = 0.06 (one-tailed).

#### Error rates

Analogous analysis on error rates revealed no significant results except for the main effect of display type, *F*(1, 12) = 6.7, *p* < 0.05, $\eta_{\text{P}}^{\text{2}}=\text{0}\text{.36,}$ showing that more errors were committed in target present trials (*M* = 3.6%, SEM = 0.8) as compared to target absent trials (*M* = 1.4%, SEM = 0.4), which suggests that participants adopted a rather conservative strategy in the visual search task by avoiding committing false alarms. Lack of congruency effects for error rates parallels previous results (Wykowska et al., 2009, 2011) and might be due to an overall small error rate (<7%).

### Event-related potentials

**Figure A1:**
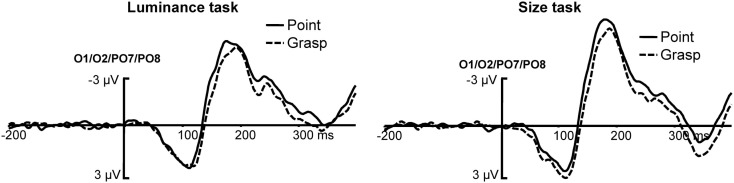
**Grand average ERP waveforms for target absent trials in luminance task (left) and size task (right)**. Solid line represents the pointing condition while the dashed line depicts the grasping condition.

As the action-related effects on visual search task were observed in RTs for target present trials and not target absent trials, the ERP analyses focused on target present trials (see Figure A1 in Appendix for analyses of target absent trials). Catch trials (no search display; movement only trials) were subtracted from “actual” trials which allowed for elimination of overlapping potentials related to presentation of the movement cues and for the extraction of potentials related to search display presentation. The subtraction was conducted on epoched data, separately for each cue type, time locked to search display onset.

#### Early sensory ERP components

**Figure 5:**
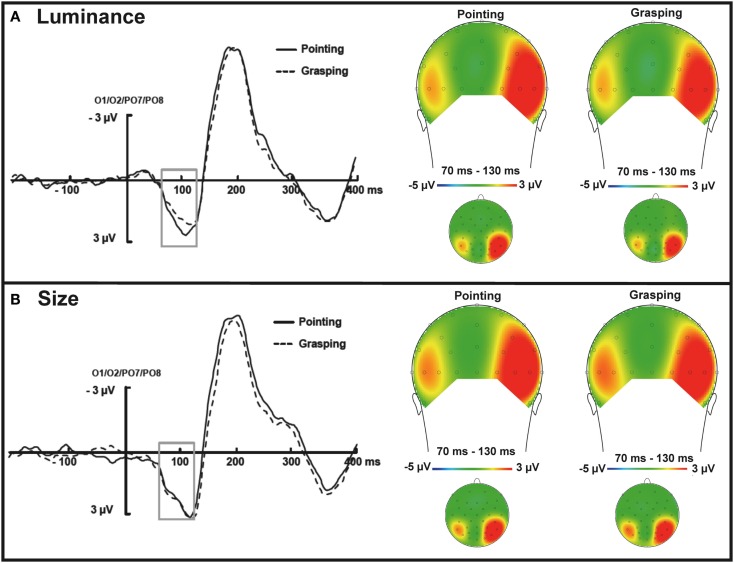
***Left*: Grand average ERP waveforms within the P1 time window of 70–130 ms for luminance targets (A) and size targets (B); targets presented in the left and right visual hemifield are averaged together**. The grand average waveforms are pooled across occipital electrodes (O1/O2 and PO7/PO8) locked to the search display. Solid lines represent the pointing movement condition whereas the dashed lines represent the grasping condition. Gray outline boxes indicate the P1 effect (70–130 ms) for luminance targets and lack thereof for size targets. *Right*: Topographical maps of voltage distribution for the same time intervals, presented from posterior view (larger images) and top view, all channels (smaller images, front plotted upwards). Note that the scalp distribution of the mean amplitude within the P1 component time window indicates a larger positivity on the right electrode sites, independent of condition. This might be related to the fact that attentional networks are located mostly in the right cerebral hemisphere (e.g., Heilman and Van Den Abell, 1980; Mesulam, 1981; Sperry, 1974; Thiebaut et al., 2011), and is in line with previous findings on attentional orienting that showed validity effects in a cueing paradigm also predominantly on right lateral electrodes (e.g., Mangun and Hillyard, 1991).

A 2 × 2 × 4 ANOVA with the factors task type (size vs. luminance), movement type (grasping vs. pointing) and electrode (O1, O2, PO7, PO8) conducted on the mean amplitudes of the ERP waveform within 70–130 ms time window [representing the latency of the P1 component, determined around (±30 ms) the grand average peak latency] revealed a significant interaction of task type and movement type, *F*(1, 13) = 6.2, *p* < 0.05, $\eta_{\text{P}}^{\text{2}}=\text{0}\text{.32}$ indicating a more enhanced positivity for the pointing movement (*M* = 1.9 μV, SEM = 0.6) relative to grasping (*M* = 1.5 μV, SEM = 0.7) in the luminance condition but not in the size condition (pointing: *M* = 2.1 μV, SEM = 0.7; grasping: *M* = 2.2 μV, SEM = 0.7), see Figure 5. This effect did not interact with electrode, *p* > 0.7.

**Figure A2:**
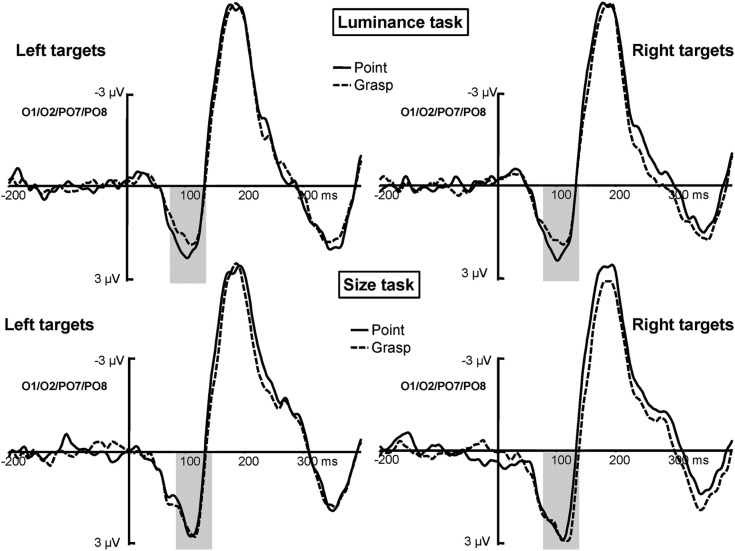
**Grand average ERP waveforms for left and right targets separately in the luminance and size task conditions**. The dark gray box depicts the P1 time window (70–130 ms) for which statistical analyses have been conducted. Solid line represents the pointing condition while the dashed line depicts the grasping condition.

The analysis for luminance and size task separately showed that this difference was indeed significant for the luminance targets, *t* (13) = 2, *p* < 0.05, one-tailed (Figure 5A) but not for size targets, *p* > 0.25, one-tailed (Figure 5B). As such, the behavioral congruency effect for luminance dimension was reflected in a P1 modulation in the ERPs. The scalp distribution of the mean amplitude of the ERPs within the 70–130 ms time window (P1) is shown in Figure 5, right. See Figure A2 in Appendix for separate analyses of trials in which targets were presented in the left vs. right hemifield.

#### Attention-related ERP – the N2pc

**Figure 6:**
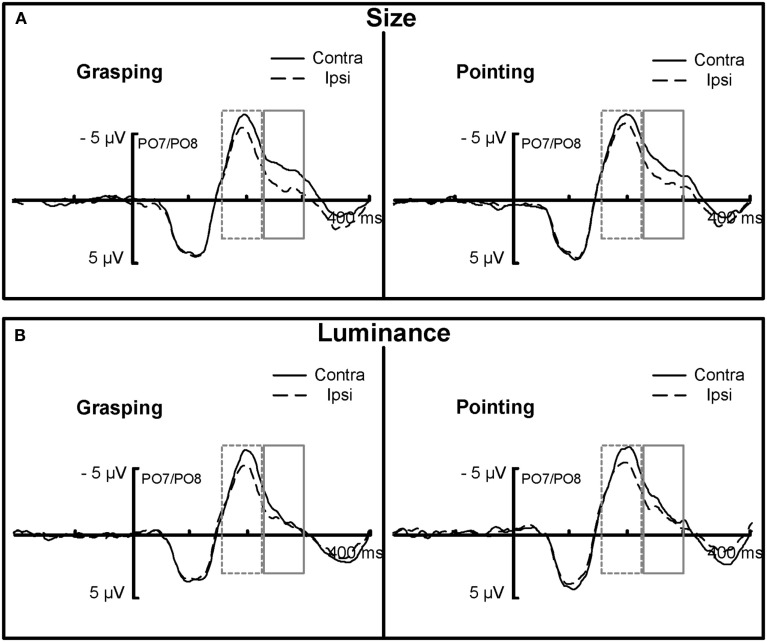
**Grand average ERP waveforms, locked to the search display plotted for ipsilateral (dashed lines) and contralateral (solid lines) electrode sites separately, pooled across PO7/PO8 electrodes for size (A) and luminance (B) targets separately as a function of grasping (left) and pointing (right) conditions**. The difference between the contralateral and ipsilateral curves, at around 180–300 ms indicates the N2pc. Solid gray boxes mark the time window (230–300 ms) in which an action-related modulation was observed for size targets: N2pc was larger for size-and-grasping as compared to size-and-pointing. No such differential effect was observed for luminance targets in this time window. The dashed boxes represent the earlier time window in which a general N2pc was observed for both luminance and size targets.

**Figure A3:**
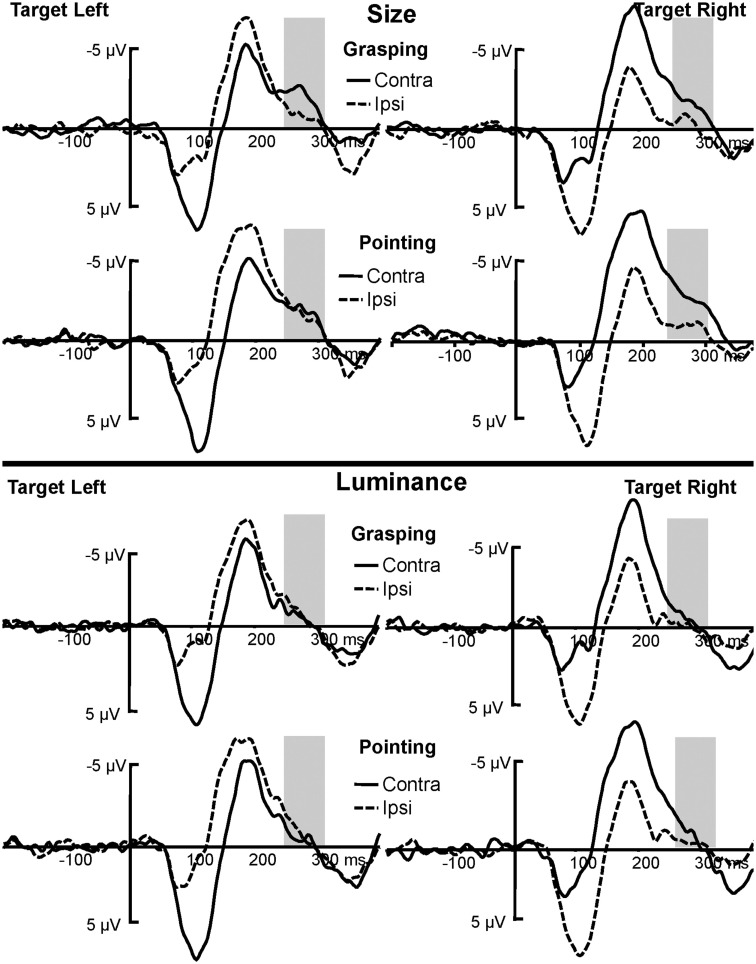
**The N2pc for left and right targets separately for PO7/8 electrode pair**. In the size and luminance tasks and grasping and pointing condition, respectively. The dark gray box depicts the N2pc time window (230–300 ms) for which statistical analyses have been conducted. Please note that PO8 is the contralateral electrode for targets on the left (solid lines in the graphs on the left), while it is ipsilateral for the targets on the right (dashed lines in the graphs on the right side). Therefore the visible difference between contra- and ipsilateral waveforms on the P1 and N1 components is due to more positive amplitude on the PO8 electrode, independent of condition. This might be related to attentional networks being located in the right cerebral hemisphere (e.g., Heilman and Van Den Abell, 1980; Mesulam, 1981; Sperry, 1974; Thiebaut et al., 2011).

In order to investigate the congruency effects on the lateralized N2pc component, the EEG signal was epoched separately for left and right targets for the PO7/PO8 electrode pairs. Subsequently, the left/right targets were averaged together for respective ipsi- and contralateral electrodes resulting in two waveforms (contralateral vs. ipsilateral) for each of the task types and movement types (see Figure A3 in Appendix for separate analyses of left- and right hemifield targets, which reveal that N2pc was not modulated by hemifield of presentation and thus left- and right targets were averaged together for the analysis of interest). A 2 × 2 × 2 (ANOVA) was performed on the N2pc mean amplitudes obtained in the 230–300 ms time window, around (±35 ms) the grand average peak latency of the difference wave between contra and ipsilateral channels with the factors laterality (contralateral vs. ipsilateral), task type (size vs. luminance), and movement type (grasping vs. pointing) for the electrode sites PO7/PO8 (Figure 6, solid boxes).

**Figure 7:**
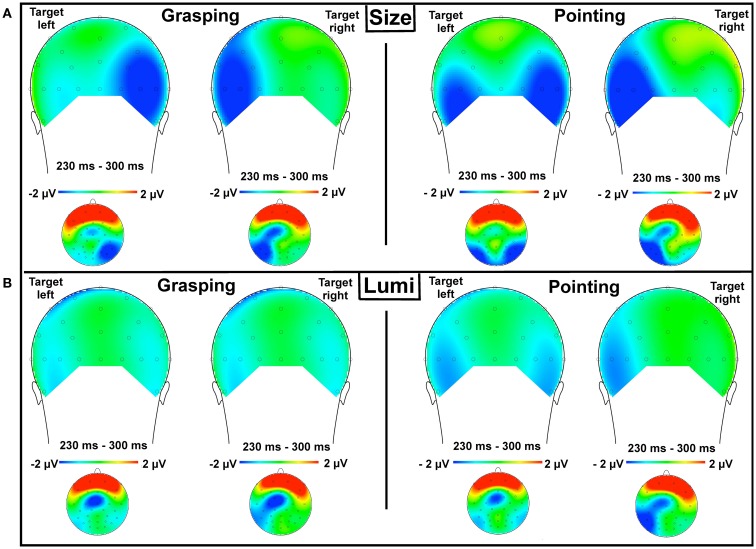
**Topographical maps of the ERP voltage distribution for the N2pc time window (230–300 ms) for size targets (A) and luminance targets (B) in the grasping condition (left) and pointing condition (right) presented from posterior view (larger images) and top view, all channels (smaller images, front plotted upwards)**. The voltage distribution maps represent un-subtracted waveforms in the respective conditions for targets presented in the left and right visual hemifields. The maps show clear target-related laterality effects (that is, enhanced activity contraleratal to the target: the N2pc) for size targets in the grasping condition [**(A)**, left], while laterality was present but less pronounced in the pointing condition [**(A)**, right]. The enhanced negativity on the ipsilateral side for the left size targets in the pointing condition [**(A)**, right] might be a slight indication of not entirely successful disengagement of attention from the right visual hemifield when targets were presented on the left. This may have resulted from the pointing cue that may have acted as a directional cue to the right hemifield. This effect, however, needs to be taken with caution, as it has not been supported by the behavioral results, see Appendix. In the luminance condition **(B)**, negativity was less pronounced in the grasping condition compared to pointing. In grasping trials, there was no difference in negativity for contra- and ipsilateral sites **(B)**, left) yet a slight difference is observed in the pointing condition for target presented in the right hemifield [**(B)**, right].

The analysis showed a main effect of laterality, *F*(1, 13) = 5.3, *p* < 0.05, $\eta_{\text{P}}^{\text{2}}=\text{0}\text{.3,}$ an interaction of laterality and task type, *F*(1, 13) = 10, *p* < 0.01, $\eta_{\text{P}}^{\text{2}}=\text{0}\text{.4,}$ and most importantly, an interaction of laterality, task type, and movement type, *F*(1, 13) = 4.5, *p* = 0.05, $\eta_{\text{P}}^{\text{2}}=\text{0}\text{.26,}$ see Figure 6. The interaction of laterality and task type showed that in this time window, N2pc was more pronounced for size targets (contralateral: *M* = −2.5 μV, SEM = 1; ipsilateral: *M* = −1.2 μV, SEM = 0.9) than for luminance targets (contralateral: *M* = −1.2 μV, SEM = 0.8; ipsilateral: *M* = −0.8 μV, SEM = 0.7). Therefore, subsequent analyses were conducted separately for each task type. The analysis on size targets revealed a main effect of laterality, *F*(1, 13) = 9, *p* < 0.05, $\eta_{\text{P}}^{\text{2}}=\text{0}\text{.4,}$ and a significant interaction of laterality and movement type, *F*(1, 13) = 5.2, *p* < 0.05, $\eta_{\text{P}}^{\text{2}}=\text{0}\text{.28}$ indicating that the N2pc was more pronounced in the grasping condition (contralateral: *M* = −2.4 μV, SEM = 1.2; ipsilateral: *M* = −0.9 μV, SEM = 0.9, see Figure 6A, left, solid gray box) as compared to pointing (contralateral: *M* = −2.6 μV, SEM = 1; ipsilateral: *M* = −1.5 μV, SEM = 0.8, see Figure 6A, right, solid gray box). Scalp distribution of the ERP waveforms in the N2pc time window of 230–300 ms for size targets in the grasping (congruent) and pointing (incongruent) conditions, separately for targets presented in the left and right visual hemifields is shown in Figure 7A.

For luminance targets, no effects reached the level of significance, all *p* > 0.15 (see Figure 6B, solid gray box). Scalp distribution of the ERP waveforms in the N2pc time window of 230–300 ms for the luminance targets in the grasping (incongruent) and pointing (congruent) conditions separately for targets presented in the left and right visual hemifields is shown in Figure 7B. Note that no clear difference in negativity was observed for contralateral and ipsilateral sites for luminance targets in the incongruent movement condition (grasping, left lower part of the Figure 7) while a slight difference (statistically non-significant) is visible in the congruent condition (pointing), targets presented in the right hemifield (lower right part of Figure 7).

As no pronounced N2pc was observed for the luminance targets in the time window of 230–300 ms, an additional analogous analysis was performed in the earlier time window of 160–230 ms (see Figure 6, dashed boxes) with the factors laterality, task type and movement type. This analysis revealed a main effect of laterality, *F*(1, 13) = 11, *p* = 0.01, $\eta_{\text{P}}^{\text{2}}=\text{0}\text{.45}$ (contralateral: *M* = −5.2 μV, SEM = 1.1; ipsilateral: *M* = −4.2 μV, SEM = 0.9), and no interactions with task type or movement type, all *p* > 0.6. This effect indicated a pronounced N2pc for both size and luminance targets in this earlier time window but no modulation thereof by movement type.

#### Control experiment

In order to examine if the action-perception congruency effects are indeed due to action preparation and not a result of mere perceptual priming related to the pictorial cues themselves, we conducted an experiment in which participants (17 in total, 7 women, mean age: 23.2; age range: 20–28) were asked to perform a visual search task for size or luminance, with the visual search displays following photographs depicting either a pointing or a grasping movement. In this experiment, however, no movement execution was required. Otherwise, the design remained identical to that of Experiment 1. The experiment consisted of 384 trials, with 192 trials in which participants searched for luminance, and 192 trials in which size was the target-defining dimension. Target dimensions were blocked, and the order was counterbalanced across participants. Pictures of a grasping or pointing movement were randomized within each block of trials. Data of 3 participants were excluded from further analyses due to large error rates (>20%). Mean RTs were subject to statistical analyses after exclusion of erroneous trials and trials on which RT exceeded ±3 SD from the mean of each participant and each task separately.

An ANOVA with the within-participants factors of *display type* (target present vs. target absent), *task type* (luminance vs. size), and *picture type* (grasping vs. pointing) as well as a between-subjects factor of *task order* (luminance first vs. size first) revealed no significant interaction of task type and picture type, *F* < 2.7 *p* > 0.12, and no significant interaction of task type, picture type and display type, *F* < 1 *p* > 0.35. When only target trials were analyzed, also no interaction between task type and picture type was observed, *F* < 0.35, *p* > 0.59, and so was the case for target absent trials, *F* < 2.5, *p* > 0.15. Finally, task order had no effect on any other effects or interactions of interest, all *p*s > 0.35.

## Discussion

The aim of this study was to investigate electrophysiological correlates of the intentional weighting mechanism observed in the form of action-perception *congruency* *effects* (Wykowska et al., 2009): better performance in search for size targets when a grasping movement was prepared as compared to a pointing movement; and better detection of luminance targets when pointing, as compared to grasping. In the present experiment, we replicated the behavioral congruency effects for target present trials. Lack of effects in target absent trials might indicate that intentional weighting operates on perceptual processing more prominently when a given signal (size or luminance) is present or when attention is more focused. In general, it is not surprising to observe different effects for target present and absent trials in a visual search task (see Chun and Wolfe, 1996 for discussion on differential processing of target present and absent trials, as well as Schubö et al., 2004b, 2007). The control experiment, in which pictures of movement cues and visual search displays were presented, but no movement was required, confirmed that the observed congruency effects are indeed due to action preparation. The fact that in this control experiment the interaction between picture type and task type was neither observed for target present nor target absent trials indicates that congruency effects do not result from some sort of low-level sensory priming related to the cue stimuli.

Importantly for the aims of the present study, we observed a modulation of early visual ERPs and the N2pc that was related to action intentions. These effects were in line with our hypotheses: if action planning biases processing of perceptual dimensions through intentional weighting, it should be possible to observe such weighting effects on pre-selective processes, reflected by P1 or N1 and, as a consequence, on attentional selection, as mirrored by the N2pc. Interestingly, we observed intentional weighting effects on early sensory P1 component (70–130 ms) for luminance targets whereas for size targets, this effect was reflected in a modulation of attention-related N2pc (230–300 ms).

More specifically, for luminance targets the P1 was more positive in the pointing movement condition relative to grasping (see Figure 5A) while for size targets there was no differential effect on P1 (see Figure 5B). The opposite pattern of results was observed for N2pc in the 230–300 time window: for size targets, the N2pc was larger in the grasping condition relative to pointing (see Figure 6A, left) and there was no effect for the luminance targets (see Figure 6B).

Interestingly, for luminance targets no pronounced N2pc was observed in this time window. It might be the case that the action-related bias of perception and attention is observable on those stages of processing that are more crucial for successful completion of a task. That is, if the task requires focal attention, then the effects might be better observed as modulation of focal attention. Similarly, if a task can be completed with mere detection of salience signals, then effects of intentional weighting can be observed already on sensory stages of processing. As behavioral results revealed that size targets were more difficult to detect than luminance targets, search for size might have been less efficient (see Wolfe, 2003 for a discussion on search efficiency). Thus, to detect size targets, more attentional focus might have been required, and hence intentional weighting effects were observed on the attention-related ERP (N2pc). On the contrary, luminance target might have been detected only based on their saliency signal and hence the stage of attentional focusing might have been less pronounced in solving the luminance detection task. Hence, in case of luminance targets, intentional weighting could be observed at the earlier ERP component, namely the P1.

### Theoretical considerations

Results of the present study support the idea that perceptual processes can be biased by action planning – an idea that has been put forward within the TEC and supported empirically (Müsseler and Hommel, 1997; Craighero et al., 1999; Hommel et al., 2001; Fagioli et al., 2007; Wykowska et al., 2009 and many others).

According to TEC, perception and action share a common representational code, which entails bi-directional influences between perception and action planning. To date, such influences have been observed mostly behaviorally (Müsseler and Hommel, 1997; Craighero et al., 1999; Bekkering and Neggers, 2002; Schubö et al., 2004a; Fagioli et al., 2007; Wykowska et al., 2009), although several studies have been conducted with neuroimaging techniques (e.g., Grafton et al., 1997; Grèzes and Decety, 2002; Schubotz and von Cramon, 2002; Handy et al., 2003). Moreover, existing research with the EEG/ERP method has shown that an ERP correlate of deviance detection (P3a) was modulated by participants’ anticipatory mechanisms related to acquired links between certain actions and their perceptual consequences (Waszak and Herwig, 2007); or that action observation influenced the N2 component dependent on whether the performed action was compatible with the observed one or not (Press et al., 2010). Action observation was shown to affect even earlier ERP components when action observation was congruent with prepared action (Bortoletto et al., 2011). Furthermore, results showed that the N1 component was modulated by action-object congruency when participants judged whether objects were real or not (Humphreys et al., 2010); or that spatial attention was shifted to the side where movement was being prepared (Eimer et al., 2005).

However, the present study is the first to show ERP correlates of a more general mechanism that biases perceptual processing toward those perceptual characteristics that can potentially be action-relevant – the *intentional weighting* mechanism. Therefore, the present results extend earlier findings of action-related bias on perception of *action*/action observation (Press et al., 2010; Bortoletto et al., 2011); and they also go beyond the idea of selection-for-action in a spatial manner (e.g., Rizzolatti et al., 1994; Deubel and Schneider, 1996). Deubel and Schneider, for example, showed that perceptual processing is facilitated in the position toward which a saccade is planned, even before the saccade is executed. In a similar line, Rizzolatti postulates the idea of premotor attention. According to Rizzolatti, spatial attention is a consequence of neuronal activity related to preparation of goal-directed, spatially organized movements.

Although other researchers have already investigated the effects of action-perception links on feature- or dimension-based selection (e.g., Craighero et al., 1999; Fagioli et al., 2007; Wykowska et al., 2009), the present results indicate the ERP correlates of such an action-related intentional weighting mechanism that operates on perceptual dimensions. It is important to note that although the action-related modulations were found on an ERP component that reflects spatial attention, the modulation was not spatial in nature. That is, the *type* of action (grasping or pointing) modulated spatial attention, and not the location of an eye or arm movement. Therefore, modulation of an ERP marker of spatial attention (N2pc) might have been a consequence of an earlier weighting mechanism that weighs perceptual dimensions according to their (action) relevance. This suggestion is plausible especially due to the fact that we observed also action-perception links imposing bias on perceptual processing at even earlier stages than allocation of spatial attention. That is, action-related effects were observed earlier than the N2pc: already at around 70 ms post-stimulus presentation. This effect is in line with the postulates put forward in Wykowska et al. (2009) as well as Hommel (2010) stating that action planning influences perception through *intentional weighting* (Hommel et al., 2001; Hommel, 2010) which operates at the level of perceptual dimensions and biases neural responses toward dimensions that are potentially action-relevant. In the case of the present experiment, luminance dimension was weighted higher for pointing actions whereas size dimension was prioritized for grasping.

We postulate that the intentional weighting mechanism is similar to other task-related biasing mechanisms (e.g., Eimer and Kiss, 2008; Lien et al., 2008; Zhang and Luck, 2009; Töllner et al., 2010; see also Desimone and Duncan, 1995; Reynolds et al., 1999 as well as Bundesen, 1990; Müller et al., 2003, or Wolfe et al., 2003 for non-ERP research on mechanisms that bias visual perception) as it is not dependent on spatial- or action-compatibility (Wykowska et al., 2011). In line with Hommel (2010), we believe that intentional weighting is a mechanism that originally developed in order to provide information for open parameters of online action control. Hence, the function of attention is not to reduce the abundance of input for further processing that has limited capacity (e.g., Broadbent, 1958; Kahneman, 1973), but rather to provide parameters for online adjustment in action control. In particular, Hommel specifies two processing pathways in action planning: an offline pathway where invariant characteristics of an action are planned and an online pathway in which particular variable parameters of a given action are specified (a particular size or location of an object). The original function of attention, therefore, has been to prepare the system for delivering the online parameters. This idea is supported by the present data, which show that attentional processes, as measured by the N2pc, are tuned to intended actions.

Finally, the observation that action-related influences reach early stages of processing is an important result, given how far action planning brain areas, i.e., premotor areas, supplementary motor areas (preSMA), parietal areas (intraparietal sulcus), and cingulate cortex (e.g., Rizzolatti et al., 1998; Rizzolatti and Luppino, 2001; Mueller et al., 2007), are located from the early visual areas, i.e., extrastriate cortex – which is claimed to be the source of the P1 component (e.g., Luck et al., 2000). Therefore, results of the present study support the idea of broad interactions between various brain regions, including action-related, and visual areas and far-reaching connections.

The present findings may also be discussed in relation to the attentional sensitization model (Kiefer and Martens, 2010; Martens et al., 2011; Kiefer, 2012), which was developed to account for various top-down controlled influences on unconscious information processing. This model claims that task representations configure the cognitive system in such a way that processing streams are modulated (“sensitized”) in accordance with the respective active task set. Attentional sensitization is supposed to enhance the sensitivity of task-relevant and attenuate the sensitivity of task-irrelevant pathways. In their experiments, Kiefer and colleagues used an induction task (either semantic or perceptual stimulus classification) in combination with a masked priming task. The induction task was presented before the masked prime and was supposed to activate either a semantic or a perceptual task set. Results showed that processing of the prime presented after the induction task was modulated by the nature of the activated task set: processing of an unconsciously perceived word prime was enhanced after a semantic induction task but not after a perceptual induction task and vice versa. Thus prime processing benefited from previous sensitization when the priming task matched the pathways sensitized by the induction tasks (Kiefer and Martens, 2010; Martens et al., 2011; Kiefer, 2012). Although there are some differences between the induction task and the present paradigm, the attentional sensitization model may also be used in order to describe the present findings. In the context of the present experiment, one may assume that action planning, which was induced by the presentation of the action cue, was accompanied by the activation of a respective task set. This task set may have modulated processing within the respective perceptual pathways. Thus a grasping cue may have modulated (“sensitized”) the action-congruent perceptual size pathway while the pointing cue may have done so for the luminance pathway. Thus, similar to differential sensitization within the perceptual domain in the experiments by Kiefer (2012), the action cue may have differentially modulated the sensitivity of size and luminance processing in the search task of the present experiment.

## Conclusion

In summary, results reported in this study revealed that visual perception and selection are influenced by action intentions. That is, what we humans focus on – among the abundance of input reaching the sensory apparatus – is already biased by how we intend to act. Through a life-long experience with our actions, we have learned what perceptual characteristics are important for a given action type. Therefore, when planning to act in that particular way, we tune our perception to what is action-relevant. This mechanism needs to be taken into account in research on selection mechanisms that is usually conducted in artificial laboratory setup. In other words, one needs to remember that people select not only what is asked from them to select but also what is related to how they intend to act.

## Conflict of Interest Statement

The authors declare that the research was conducted in the absence of any commercial or financial relationships that could be construed as a potential conflict of interest.

## Appendix

### Target absent trials

In order to test whether the different movement types of pointing vs. grasping affected the P1 in any systematic fashion during the target absent trials, we conducted a 2 × 2 × 4 ANOVA with the factors *movement type* (grasping vs. pointing), *task* *type* (luminance vs. size), and *electrode* (O1, O2, PO7, and PO8) on target absent trials in the time window of the P1 (70–130 ms). This was done to ensure that the ERP effects in the target present trials truly reflected interactions of intentions and target processing rather than a general influence of intentions. This ANOVA could only be conducted for the P1 because the N2pc is always calculated relative to the target position. The analysis showed no main effect of movement type or interaction of movement type and task type, all *F*s < 1.3, all *p*s > 0.23, see Figure A1 in Appendix. There was only a significant interaction of electrode, and movement type, *F*(1.3, 16.9) = 6.9, *p* < 0.02. Subsequent analyses for each of the electrodes showed that the effect of movement type was significant only on the PO7 electrode, *F*(1, 13) = 5.3, *p* < 0.05, $\eta_{\text{P}}^{\text{2}}=\text{0}\text{.3}$ with grasping movement eliciting more positive amplitude (*M* = 2.1, SEM = 0.8) than the pointing movement (*M* = 1.5, SEM = 0.8). This positivity effect of intention to grasp on the P1 cannot account for the strong overall P1 effect at all electrodes that was found in the analysis of the target present trials, i.e., the effect of more enhanced P1 for pointing vs. grasping in the luminance task condition.

In a subsequent time window (130–300 ms) there was a main effect of movement type *F*(1, 13) = 12.5, *p* < 0.01, $\eta_{\text{P}}^{\text{2}}=\text{0}\text{.5}$ with grasping movement eliciting less negative amplitude (*M* = −2.3, SEM = 0.9) than the pointing movement (*M* = −3, SEM = 0.8), all other effects and interactions were non-significant, all *F*s < 0.9, all *p*s > 0.4.

More enhanced negativity for the pointing condition in target absent trials, independent of the task type might indicate that when there was no signal to be processed in the visual search display, more neuronal resources were employed when a pointing movement was prepared, relative to a grasping movement. This might be due to pointing being in general a simpler movement than grasping. Hence, more resources could have been devoted to the visual search task. This interpretation, however, needs to be taken with caution, as no corresponding effect was observed in behavior (main effect of movement type in target absent trials was not significant, *F* < 1.5, *p* > 0.25). Importantly, the differential effect of movement type in target absent trials was observed for a different time window than the effect of interest (P1) observed in target present trials in luminance task.

### Catch trials

In order to examine the ERPs in the catch trials, i.e., baseline trials, which were subtracted from the trials of interest, we conducted a 2 × 4 ANOVA with the factors *movement type* (grasping vs. pointing) and *electrode* (PO7, PO8, O1, O2) over the time window of the P1 component (70–130 ms). This was done in order to examine whether the effects of interest are not due to the subtraction procedure. The analysis revealed that the main effect of movement type was significant, *F*(1, 13) = 5.1, *p* < 0.05, $\eta_{\text{P}}^{\text{2}}=\text{0}\text{.28}$ with pointing movement yielding a more negative amplitude (−3.9 μV) as compared to grasping (−3 μV). This difference in the baseline trials cannot however explain the significant *interaction* between movement type and task type in the P1 time window for the trials of interest, as the very same catch trials were subtracted from the luminance and size condition.

### Left- and right hemifield of target presentation

In order to test whether the cues differentially influenced target detection on right and left sides of the visual field, we analyzed detection of right- and left-presented targets separately. Note that even though the arm presented in the cues always extended from the lower left corner of the photograph to the middle (and not to the right), attention might have been guided to search for the target first to the right side, as the arms might have acted as directional cues for attention. This could be the case for the pointing movement in particular, as an extended finger in pointing gestures is a salient directional cue.

#### P1 component

A 2 × 2 × 4 ANOVA with the factors *movement type* (grasping vs. pointing), *target position* (left vs. right), and *electrode* (O1, O2, PO7, and PO8) for the time window 70–130 ms (P1) in the *luminance* task condition showed that the main effect of movement type in the luminance condition on the P1 component was not dependent on whether the target was presented in the left or in the right visual hemifield: interaction of movement type and side of the target was not significant, *F* < 0.01, *p* > 0.9, see Figure A2 in Appendix.

#### N2pc effect

A 2 × 2 × 2 ANOVA with the factors *movement type* (grasping vs. pointing), *target position* (left vs. right), and *laterality* (contra- vs. ipsilateral) for the time window of 230–300 ms (N2pc) in the *size* condition showed that the side in which target was presented had no general effect on the N2pc, interaction of laterality and target position, *F* < 0.05, *p* > 0.58. Only the interaction of laterality and movement type slightly depended on the hemifield in which the target was presented: interaction of laterality, movement type and side of target was marginally significant, *F*(1, 13) = 3.6, *p* = 0.08, $\eta_{\text{P}}^{\text{2}}=\text{0}\text{.2,}$ see Figure A3 in Appendix. For targets presented in the left hemifield, the interaction of laterality and movement type was significant, *F*(1, 13) = 7.25, *p* < 0.02, $\eta_{\text{P}}^{\text{2}}=\text{0}\text{.36}$ while for targets presented in the right hemifield this interaction was not significant, *F* < 0.4, *p* > 0.54. Analogous analysis for the luminance targets showed no significant effects or interactions, all *F*s < 2.8, *p*s > 0.12. More pronounced movement-related effect on N2pc for targets presented in the left hemifield speaks against the interpretation that the effect is due to cues guiding attention to the right side of the visual field. Rather, this might be due to preferential processing of the left hemifield related to attentional networks being located in the right cerebral hemisphere (please see below). Alternatively, analyzing left- and right hemifield targets separately might have reduced the statistical power, and hence the effects reached the level of significance only in one of the conditions.

### Behavior

An additional analysis on behavioral data in target present trials with the factors *movement type* (grasping vs. pointing), *target position* (right vs. left), and *task type* (luminance vs. size) showed that there was no main effect of target position, *F* < 0.05, *p* > 0.85, no interaction of movement type and target position, *F* < 0.05, *p* > 0.85, and revealed also that the interaction of interest (movement type and task type) did not depend on the hemifield in which the target was presented, *F* < 1, *p* > 0.3.

Also in the control experiment, an analogous ANOVA with the factors *picture type* (grasping vs. pointing), *target position* (left vs. right), and *task type* (luminance vs. size) revealed no significant effect of target position, *F* < 1.5 *p* > 0.25; no interaction of picture type and target position, *F* < 0.05, *p* > 0.8 and no interaction between picture type, target position, and task type *F* < 0.6, *p* > 0.45.

Taken together, the results of separate analyses for trials in which targets were presented in the left vs. right hemifields indicate that targets presented in the right hemifield were not processed preferentially. Therefore, the cues have not cued participants’ attention to the right side of the visual field, which could potentially influence the congruency effects.
